# HSPG2 could promote normal haematopoiesis in acute myeloid leukaemia patients after complete remission by repairing bone marrow endothelial progenitor cells

**DOI:** 10.1002/ctm2.70220

**Published:** 2025-02-06

**Authors:** Chen‐Yuan Li, Zhen‐Kun Wang, Tong Xing, Meng‐Zhu Shen, Xin‐Yan Zhang, Dan‐Dan Chen, Yu Wang, Hao Jiang, Qian Jiang, Xiao‐Jun Huang, Yuan Kong

**Affiliations:** ^1^ Peking University People's Hospital Peking University Institute of Hematology National Clinical Research Center for Hematologic Disease Beijing Key Laboratory of Hematopoietic Stem Cell Transplantation Collaborative Innovation Center of Hematology Peking University Beijing China; ^2^ Peking‐Tsinghua Center for Life Sciences Academy for Advanced Interdisciplinary Studies Peking University Beijing China

**Keywords:** Acute myeloid leukemia, Complete remission, Endothelial progenitor cells, Hematopoiesis

## Abstract

**Background:**

Even after achieving complete remission (CR), many acute myeloid leukaemia (AML) patients suffer from poor haematopoietic recovery after chemotherapy. Previous studies have shown that the damage of bone marrow endothelial progenitor cell (BM EPC) hinders haematopoietic recovery after chemotherapy in mice. Therefore, elucidation of the mechanism and repair strategy of chemotherapy‐induced BM EPC damage is urgent needed.

**Methods:**

The prospective case–control study enrolled 40 AML patients after CR (CR patients), who received idarubicin and cytarabine (IA) regimen (*n* = 20), or homoharringtonine, aclarubicin and cytarabine (HAA) regimen (*n* = 20) as induction chemotherapy, and their age‐matched healthy controls (HCs, *n* = 20). The HSPG2 expression level in BM EPCs and BM plasma were determined via flow cytometry and enzyme‐linked immunosorbent assays. The BM EPC's functions were evaluated by apoptosis, reactive oxygen species (ROS) level, migration and tube formation assays. The haematopoiesis‐supporting ability and leukaemia cell‐supporting ability of BM EPCs were assessed through coculture assay. Moreover, RNA sequencing and qPCR were performed to further explore the underlying mechanism.

**Results:**

HSPG2 levels decreased in both the BM plasma and BM EPCs of CR patients after IA and HAA induction chemotherapy. Moreover, the BM EPC's functions of CR patients were reduced. In vitro experiments demonstrated that the *HSPG2* gene knockdown or cytosine arabinoside treatment led to BM EPC dysfunction, whereas the HSPG2 treatment promoted repair of the BM EPC function in vitro. In addition, we found that the HSPG2 treatment restored the BM EPC function from CR patients without affecting their leukaemia cell‐supporting ability. Mechanistically, BM EPC functions and haematopoietic regulation‐related genes were significantly decreased after the *HSPG2* gene knockdown.

**Conclusion:**

Our findings demonstrate a significant role of HSPG2 in BM EPC functions. This discovery uncovers that HSPG2 is a potential therapeutic target for promoting the BM EPC function of AML‐CR patients after chemotherapy.

**Highlights:**

- The HSPG2 level in the BM EPCs of AML‐CR patients was decreased, which was related to the reduced BM EPC function.
- *HSPG2* knockdown or Ara‐C intervention reduced the HSPG2 level and led to BM EPC dysfunction, which could be restored by HSPG2 treatment in vitro.
- HSPG2 treatment restored the BM EPC function of AML‐CR patients without affecting their leukaemia cell‐supporting ability.

## Graphical abstract

HSPG2 level in BM EPCs of AML‐CR patients was decreased, which was related to the reduced BM EPC function. *HSPG2* knockdown or Ara‐C intervention reduced HSPG2 level and led to BM EPC dysfunction, HSPG2 treatment could repair these damages and restored the BM EPC function of AML‐CR patients.

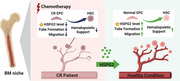

## 1 BACKGROUND

In bone marrow (BM) microenvironment, the haematopoietic process relies on haematopoietic stem cells (HSCs),^1^ where endothelial progenitor cells (EPCs), macrophages, amongst others, collaborate to control the fate of HSCs.^2^, ^3^, ^4^ Acute myeloid leukaemia (AML), a haematopoietic malignancy origins in aberrant proliferation and excessive accumulation of leukaemia cells.^5^, ^6^ AML can change the BM microenvironment into a leukaemia cells‐supporting environment, causing HSC exhaustion.^7^, ^8^, ^9^, ^10^ Chemotherapy, a type of myeloablative therapies, is the major treatment strategies.^11^ Even after achieving complete remission (CR), many AML patients suffer from poor haematopoietic recovery after chemotherapy, which is characterised by an increased risk of anaemia, bleeding and infection.^12^ Therefore, exploration of the mechanism of poor haematopoietic recovery after chemotherapy is important.

Accumulating evidence has indicated that bone marrow endothelial progenitor cells (BM EPCs) play invaluable roles in regulating the haematopoietic process.^3^, ^13^ Our previous works have shown that impaired BM EPCs are related to the occurrence of haematopoietic dysfunction‐related disorders, for example, poor haematopoietic function after chemotherapy or transplantation, myelodysplastic neoplasms or aplastic anaemia, and repairing dysfunctional BM EPCs could promote haematopoiesis in these diseases.^14^, ^15^, ^16^, ^17^, ^18^, ^19^, ^20^, ^21^, ^22^, ^23^ In mice model, previous researches showed that BM EPC damage delays the recovery of haematopoiesis after chemotherapy.^21^, ^24^, ^25^ Therefore, identifying the mechanism and repair strategy for promoting BM EPC function in AML‐CR patients are of great significances.

*Heparan sulphate proteoglycan 2* (*HSPG2*) gene, whose expression influences many processes including the formation of multiple organs.^26^ The protein encoded by this gene, also known as perlecan, a large multidomain extracellular matrix (ECM) proteoglycan, which could be secreted into the ECM by various cells including EPCs. In turns, HSPG2 could regulate cell proliferation and differentiation.^27^, ^28^ *HSPG2* knockout mice exhibited chondrogenic‐, osteogenic‐ and adipogenic‐potential in synovial mesenchymal cells, indicating that HSPG2 is required for osteophyte formation in the knee.^29^ Research has shown that HSPG2 is essential for EPC vascularization because it modulates vascular endothelial growth factor (VEGF) signalling.^30^ In haematopoietic regulation, mutation of the *HSPG2* gene leads to impaired haematopoietic cell maturation in the lymph glands of *Drosophila*, indicating the potential role of HSPG2 in haematopoietic regulation.^31^ However, whether HSPG2 can promote the BM EPC's functions, thereby affecting the haematopoiesis process are unknown.

In this study, the HSPG2 levels and BM EPC's functions were compared between AML patients after CR (CR patients) who received idarubicin and cytarabine (IA) regimen or homoharringtonine, aclarubicin, and cytarabine (HAA) regimen as induction chemotherapy and their age‐matched healthy controls (HCs). To clarify the effect of chemotherapy on BM EPCs, we established an in vitro chemotherapy‐damage and HSPG2‐repairment model of BM EPCs. Moreover, the underlying mechanism was explored by RNA‐seq. We subsequently investigated whether HSPG2 can restore the BM EPC function of CR patients, especially their haematopoiesis‐supporting ability, without affecting their leukaemia cell‐supporting ability. Our goal was to elucidate the effect of HSPG2 in regulating human BM EPC functions, thus providing potential therapeutic options for AML‐CR patients after chemotherapy.

## 2 MATERIALS AND METHODS

### 2.1 Sample collection

For the prospective case–control study, BM samples of AML‐CR patients received IA regimen (CR‐IA patients, *n* = 20) or HAA regimen (CR‐HAA patients, *n* = 20) as induction chemotherapy and their HCs (*n* = 20) were included and no significant differences existed in age or sex amongst three groups (Table S1). Samples were collected after consent was obtained, these processes were in compliance with the Declaration of Helsinki, which were approved by the Ethics Committee of Peking University People's Hospital.

### 2.2 Isolation, cultivation and characterisation of bone marrow endothelial progenitor cells

BM mononuclear cells and cell cultivation were isolated as previously reported.^14^, ^15^, ^16^, ^21^, ^32^, ^33^ For identification, BM EPCs were gated by CD45^+^CD34^+^CD309^+^ cells. The data were analysed by BD FACSDiva v8.0 Software (BD Biosciences). All the antibodies are listed in Table S2.

After cultured in 6‐well plates for 7 days as previously reported,^14^, ^15^, ^16^ BM EPCs were stained with DiI‐acetylated low‐density lipoprotein (LDL) (Life Technologies, Gaithersburg, USA) for 4 h at 37°C. After fixed with prechilled paraformaldehyde and stained with 10 µg/mL Ulex europaeus agglutinin‐I (UEA I; Sigma, St. Louis, USA), the cells counted via fluorescence microscope (Olympus, Tokyo, Japan) in three random visual fields.

### 2.3 Tube formation and migration assays

4 × 10^4^ BM EPCs were plated in 24‐well Matrigel‐precoated plates as previously reported.^16^, ^21^, ^33^ Cell migration assays performed in Transwell chambers. In brief, 4 × 10^4^ BM EPCs were plated in the upper chambers, and 500 µL of the medium was added in the lower chamber, then cultured for 1 day and fixed for 15 min. 1% crystal violet was used to stain the cells on the underside of upper chambers. All assays were observed in three random fields via microscope (Olympus, Tokyo, Japan).

### 2.4 BM EPCs–HSCs, leukaemia stem cells, KG‐1 cells or HL‐60 cells in vitro coculture assay

To assess normal or malignant haematopoiesis‐supporting ability of BM EPCs, the BM EPCs in each group were cocultured with CD34^+^ cells from HCs and AML patients, KG‐1 cells or HL‐60 cells. After 1 × 10^5^ BM EPCs/well were pre‐plated on 24‐well plates for 1 day, HSCs, leukaemia stem cells (LSCs), KG‐1 cells or HL‐60 cells (1 × 10^5^ cells/well) were added and cocultured for 5 days. Apoptotic cell ratio and reactive oxygen species (ROS) level were conducted as previously reported.^14^, ^15^, ^16^

### 2.5 Colony‐forming unit and leukaemia colony‐forming unit assays

Colony‐forming unit (CFUs) assays were conducted via the Metho‐Cult H4434 method (Stem Cell Technologies, Vancouver, Canada). 2 × 10^3^ cells were cultured in 24‐well plates after coculture for 2 weeks. The method for counting the numbers of CFUs and leukaemia colony‐forming unit (CFU‐Ls) were as previously reported.^16^, ^21^, ^33^

### 2.6 5‐Ethynyl‐20‐deoxyuridine assay

The cells were stained with 50 µM ethynyl‐deoxyuridine (EdU) at 37°C for 1 h. The percentage of EdU^+^ cells was measured by BD FACSDiva v8.0 software (BD Biosciences).

### 2.7 An in vitro model of chemotherapy‐induced damage model of bone marrow endothelial progenitor cells

BM EPCs from HCs were cultivated at 37°C for 1 day in multiple concentrations medium of cytosine arabinoside (Ara‐C). For evaluation of the damage of Ara‐C to BM EPCs, tube forming experiment of BM EPCs was conducted. A 10 µM Ara‐C concentration has caused severe damage to BM EPCs and prevented the formation of tubes, so 10 µM Ara‐C was selected.

### 2.8 Transfection of bone marrow endothelial progenitor cells from healthy controls

Small interfering RNA targeting HSPG2 (sequence: 5′‐CGATGCGTCTCTACCAACA‐3′) (RiboBio, China) was ersatz. The transfection process was conducted in 2×10^6^ BM EPCs of HCs with Lipofectamine 3000 reagent (Thermo Fisher Scientific, Waltham, USA) in 6‐well plates.

### 2.9 Enzyme‐linked immunosorbent assay

After centrifuging the BM samples for 5 min at 2000 rpm, plasma on the upper side was harvested. The HSPG2 levels were measured via human HSPG2 enzyme‐linked immunosorbent assay (ELISAs) (Abcam, Cambridge, UK).

### 2.10 RNA‐seq

RNA‐seq was conducted to explore the changes in BM EPCs of HCs before and after *HSPG2* gene knockdown. Differential gene expressions (DGEs), Gene Ontology biological process (GOBP) terms and Kyoto Encyclopedia of Genes and Genomes (KEGG) enrichment plots were generated via DESeq2, cluster profiler and ggplot2 packages in R (1.16.1).

### 2.11 qRT‐PCR

The mRNA levels of *HSPG2*, *ADRA2*, *ECM1*, *ANGPT2*, *IL34*, *DCSTAMP*, *VSIR*, *INHBA* and *AXL* were determined via a qRT‐PCR kit (Thermo Fisher Scientific, Waltham, USA).^34^ The sequences of primer are shown in Table S3.

### 2.12 Statistical analysis

Data analyses were conducted by GraphPad Prism 8.0. The paired data were analysed by paired *t*‐tests and the continuous variables were analysed by Mann‒Whitney *U* tests. Results are showed by means ± standard error of the mean (SEM), *P*‐values < .05 were considered to have statistical significance.

## 3 RESULTS

### 3.1 HSPG2 levels and functions of BM EPCs decreased in AML patients after CR

**FIGURE 1:**
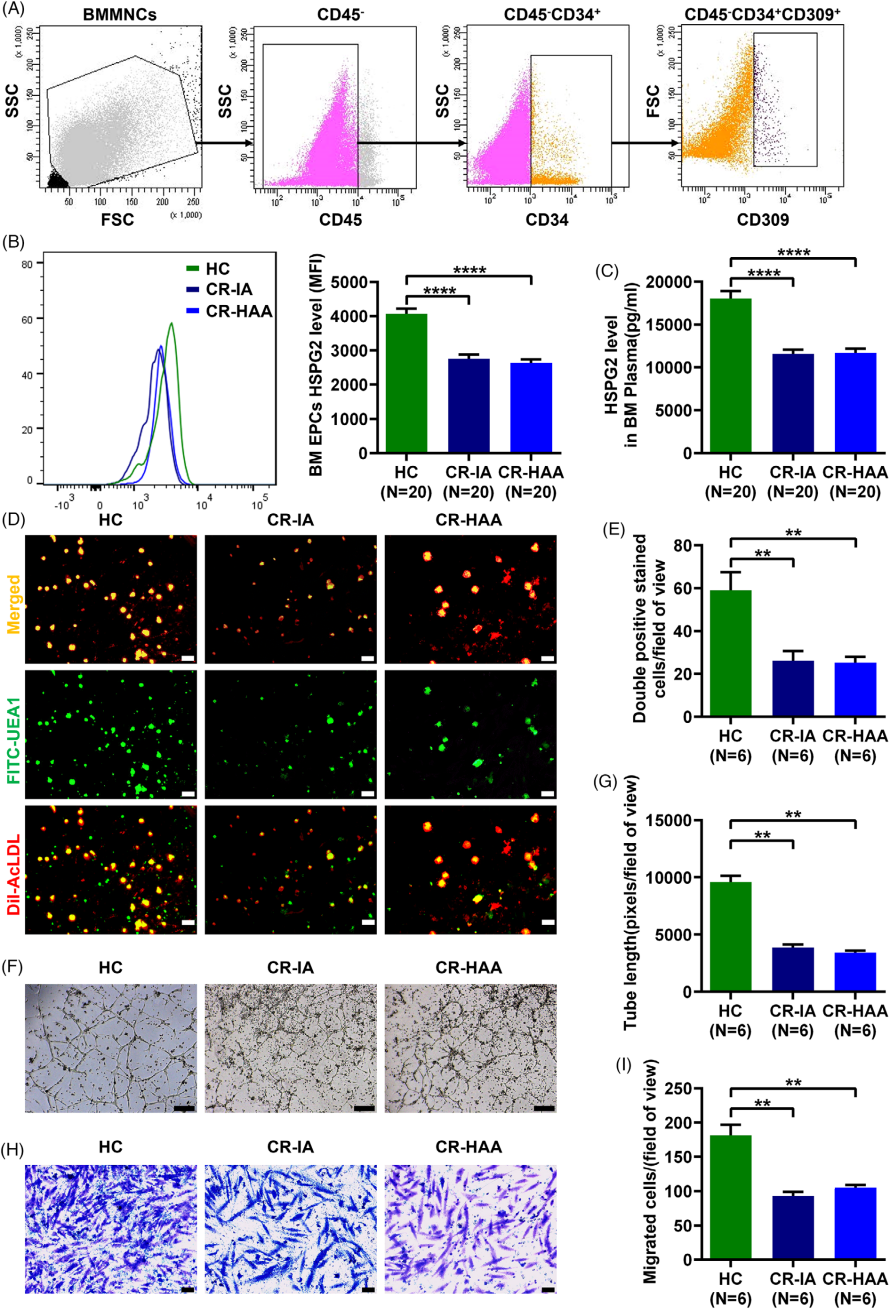
HSPG2 levels and functions of BM EPCs decreased in AML patients after CR. (A) The BM EPC phenotype was characterised by the negative expression of CD45 and the positive expression of CD34 and CD309 by flow cytometry. (B) HSPG2 levels in the gated BM EPCs from CR‐IA patients, CR‐HAA patients and HCs were analysed by flow cytometry. (C) HSPG2 levels in BM plasma samples from CR‐IA patients, CR‐HAA patients and HCs were measured by ELISA assay. (D) Representative images (scale bars represent 50 µm) of double‐positive stained with DiI‐AcLDL (red) and FITC‐UEA I (green) BM EPCs (merged in yellow)/field of view (original magnification, 10×). (E) Quantification of double‐positive stained BM EPCs/field of view (original magnification, 10×). (F) Representative images (scale bars represent 200 µm) of tube formation (pixels of tubes per field of view) by BM EPCs (original magnification, 4×). (G) Quantification of the tube length (pixels of tubes per field of view) of BM EPCs (original magnification, 4×). (H) Representative images (scale bars represent 50 µm) of migrated cells (original magnification, 10×). (I) The number of migrated BM EPCs per field of view (original magnification, 10×). The data represent the mean ± SEM. **P ≤ .01, ****P ≤ .0001. BFU, burst forming unit‐erythroid; BM EPC, Bone marrow endothelial progenitor cell; CR, complete remission; DiI‐Ac‐LDL, DiI‑acetylated low‑density lipoprotein; ELISA, enzyme‐linked immunosorbent assay; FITC‐UEA1, FITC‑labelled ulex europaeus agglutinin I; HC, healthy control; LSC, leukaemia stem cells; SEM, standard error of the mean.

The prospective case–control study was carried out to contrast HSPG2 level and BM EPC's functions between CR patients and HCs. Compared with HCs, the HSPG2 expression level in BM EPCs was significantly lower in CR‐IA patients (Figure 1A,B; 2751.0 ± 127.3 vs. 4059.0 ± 161.7, *P <* .0001) and CR‐HAA patients (Figure 1A,B; 2632.0 ± 105.0 vs. 4059.0 ± 161.7, *P <* .0001). Meanwhile, the HSPG2 level in BM plasma was significantly lower in CR‐IA patients (Figure 1C; 11560.0 ± 493.2 vs. 18016.0 ± 853.7, *P <* .0001) and CR‐HAA patients (Figure 1C; 11682.0 ± 483.3 vs. 18016.0 ± 853.7, *P <* .0001). However, there was no difference in HSPG2 levels of BM EPCs and BM plasma between the two groups of patients who received IA or HAA induction. Furthermore, there were significantly fewer double‐positive stained BM EPCs in CR‐IA patients (Figure 1D,E; 26.2 ± 4.4 vs. 59.0 ± 8.4, *P *= .004) and CR‐HAA patients (Figure 1D,E; 25.1 ± 2.8 vs. 59.0 ± 8.4, *P =* .002) than those in HCs. Compared with HCs, the tube formation ability of BM EPCs was decreased in CR‐IA patients (Figure 1F,G; 3844.0 ± 298.7 vs. 9582.0 ± 540.3, *P *= .002) and CR‐HAA patients (Figure 1F,G; 3432.0 ± 147.9 vs. 9582.0 ± 540.3, *P = *.002). Meanwhile, the migration ability of BM EPCs was decreased in CR‐IA patients (Figure 1H,I; 92.7 ± 6.1 vs. 181.3 ± 15.3, *P *= .002) and CR‐HAA patients (Figure 1H,I; 105.0 ± 3.8 vs. 181.3 ± 15.3, *P = *.002). However, there was no difference in BM EPC's functions between the two groups of patients. Thus, our findings indicate that the HSPG2 level was significantly decreased in the BM EPCs of CR patients, which may be related to the decreased quality and functions of BM EPCs.

### 3.2 *HSPG2* knockdown impaired the functions of BM EPCs from HCs, especially their haematopoiesis‐supporting ability

**FIGURE 2:**
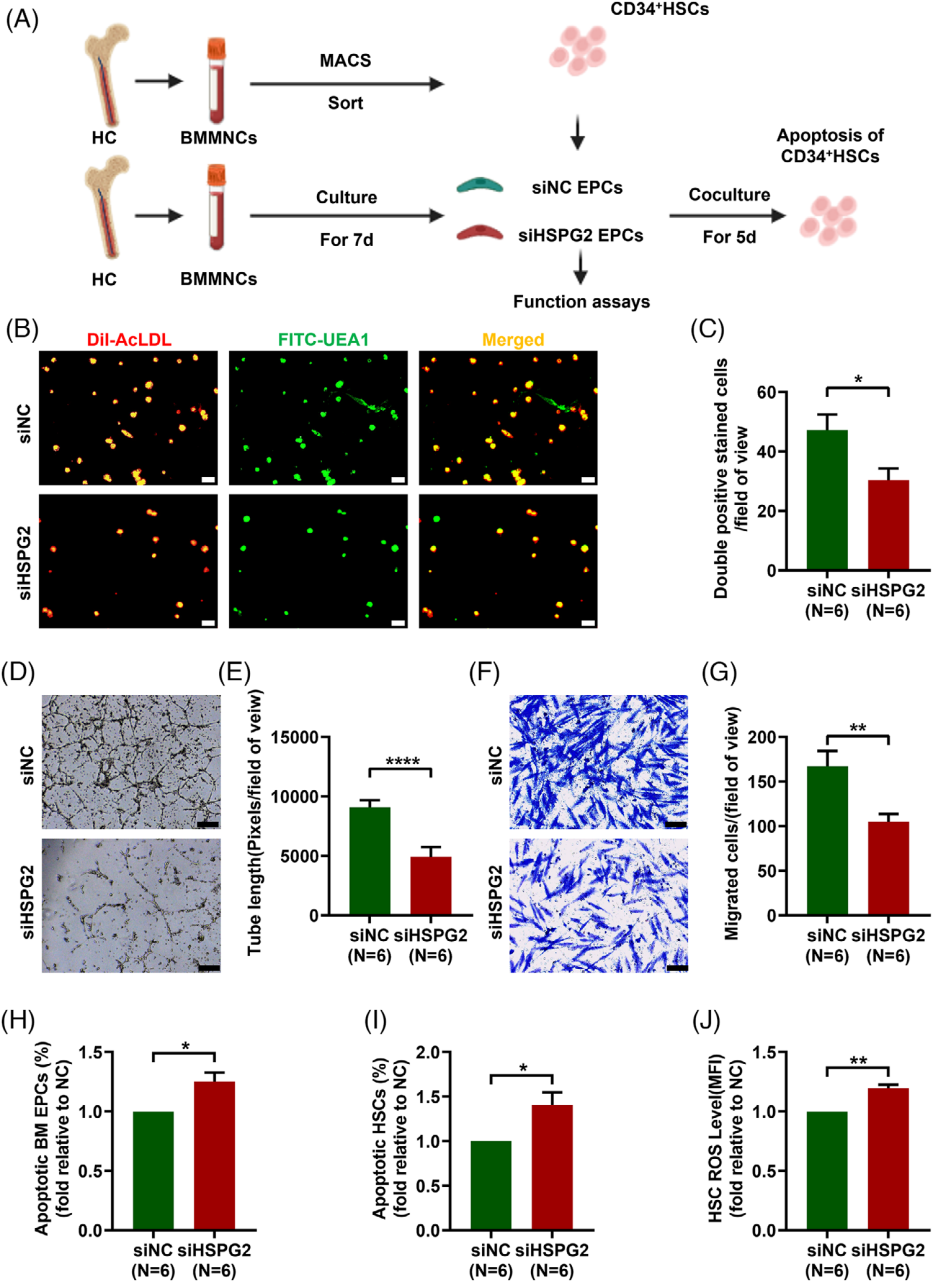
*HSPG2* knockdown impaired the functions of BM EPCs from HCs, especially their haematopoiesis‐supporting ability. (A) Schematic diagram showing the study design. Representative images (scale bars represent 50 µm) (B) and quantification (C) of double‐positive stained with DiI‐AcLDL (red) and FITC‐UEA I (green) BM EPCs (merged in yellow)/field of view (original magnification, 10×). Representative images (scale bars represent 200 µm) (D) and quantification (E) of tube formation (pixels of tubes per field of view) by BM EPCs (original magnification, 4×). Representative images (scale bars represent 50 µm) (F) and quantification (G) of migrated cells (original magnification, 10×). (H) Quantification of the percentage of apoptotic BM EPCs in the siNC and siHSPG2 groups. (I) Quantification of the percentage of apoptotic HSCs after coculture with BM EPCs. (J) Quantification of the level of intracellular ROS in HSCs. The data represent the mean ± SEM. *P ≤ .05, **P ≤ .01, ****P ≤ .0001. BM EPC, Bone marrow endothelial progenitor cell; DiI‐Ac‐LDL, DiI‑acetylated low‑density lipoprotein; FITC‐UEA1, FITC‑labelled ulex europaeus agglutinin I; HC, healthy control; HSC, haematopoietic stem cell; ROS, reactive oxygen species.

To clarify the impact of chemotherapy on the HSPG2 levels and the effects of HSPG2 levels on BM EPC functions, different concentrations of Ara‐C intervention and *HSPG2* gene knockdown assay were conducted, respectively. The pattern diagram shows the study design (Figure 2A). The relative *HSPG2* mRNA expression in BM EPCs revealed that the si*HSPG2* 2^#^ sequence had the highest knockdown efficacy (Figure S1A, 0.2 ± 0.01‐fold, *P < *.0001). After knockdown the *HSPG2* gene by 2^#^ sequence, the HSPG2 level in BM EPCs was significantly lower than the siNC group via flow cytometry (Figure S1B; 4001.0 ± 110.1 vs. 5192.0 ± 71.9, *P *= .02). Thus, the 2^#^ sequence of siHSPG2 was used for subsequent experiments. The relative HSPG2 mRNA expression level in BM EPCs was decreased as the concentration of Ara‐C increased (Figure S1C). In terms of BM EPC's quantity, the number of double‐positive‐stained cells was notably lower in the siHSPG2 group (Figure 2B,C; 30.3 ± 3.9 vs. 47.2 ± 5.2, *P *= .01). Moreover, BM EPCs in the si*HSPG2* group presented a decreased tube formation ability (Figure 2D,E; 4911.0 ± 817.5 vs. 9077.0 ± 604.2, *P <* .0001), a worse migration ability (Figure 2F,G; 104.8 ± 8.8 vs. 167.0 ± 17.2, *P *= .004) and a significantly increased apoptosis rate (Figure 2H; 1.3 ± 0.1‐fold, *P *= .031). Moreover, the siHSPG2 group showed a substantial increase in the apoptosis rate (Figure 2I; 1.4 ± 0.1%‐fold, *P *= .035) and ROS level (Figure 2J; 1.2 ± 0.03‐fold, *P *= .001) of cocultured CD34^+^ cells. Together, these data further verify that Ara‐C led to the decrease in the HSPG2 level of BM EPCs, which may be related to their impaired functions.

### 3.3 HSPG2 alleviated Ara‐C induced dysfunction in BM EPCs from HCs

**FIGURE 3:**
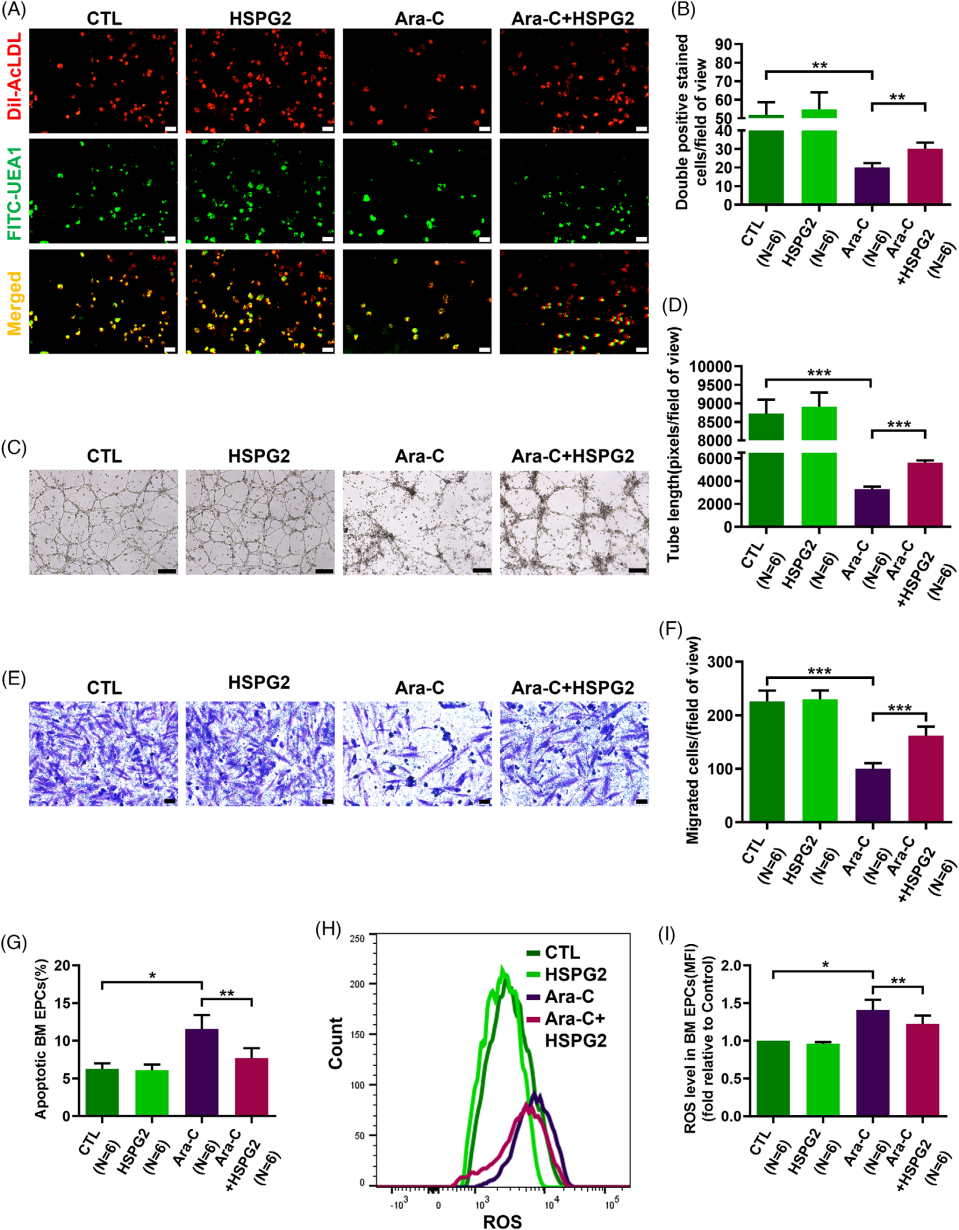
HSPG2 alleviated Ara‐C induced dysfunction in BM EPCs from HCs. To investigate the effects of chemotherapy and HSPG2 on BM EPC functions, we divided 7‐day cultivated HC EPCs into the following groups for Ara‐C and HSPG2 intervention in vitro: the control (CTL) group, the HSPG2 intervention (HSPG2) group, the Ara‐C intervention (Ara‐C) group, and the HSPG2+Ara‐C intervention (Ara‐C+HSPG2) group. Representative images (scale bars represent 50 µm) (A) and quantification (B) of double‐positive stained with DiI‐AcLDL (red) and FITC‐UEA I (green) BM EPCs (merged in yellow)/field of view in each group (original magnification, 10×). Representative images (scale bars represent 200 µm) (C) and quantification (D) of tube formation (pixels of tubes per field of view) by BM EPCs (original magnification, 4×). Representative images (scale bars represent 50 µm) (E) and quantification (F) of migrated cells (original magnification, 10×). (G) Quantification of the percentage of apoptotic BM EPCs in each group. Image (H) and quantification (I) of the ROS levels (MFIs) of BM EPCs in each group. The data are presented as the means ± SEMs. *P ≤ .05, **P ≤ .01, ***P ≤ .001. BM EPC, Bone marrow endothelial progenitor cell; DiI‐Ac‐LDL, DiI‑acetylated low‑density lipoprotein; FITC‐UEA1, FITC‑labelled ulex europaeus agglutinin I; HC, healthy control; HSC, haematopoietic stem cell; ROS, reactive oxygen species.

To explore the roles of Ara‐C and HSPG2 on BM EPCs, we divided 7‐day cultivated HC EPCs into the following groups for Ara‐C and HSPG2 intervention: the control (CTL) group, the HSPG2 intervention (HSPG2) group, the Ara‐C intervention group (Ara‐C) group and the HSPG2+Ara‐C intervention (Ara‐C+HSPG2) group. The Ara‐C treated BM EPCs presented a declined quality of double‐positive‐stained cells (Figure 3A,B; 20.0 ± 2.5 vs. 52.0 ± 6.7, *P *= .002), an impaired tube formation ability (Figure 3C,D; 3298.0 ± 221.8 vs. 8728.0 ± 372.7, *P *= .0002), a worse migration ability (Figure 3E,F; 100.2 ± 10.4 vs. 226.3 ± 19.8, *P *= .0003), increased levels of cell apoptosis (Figure 3G; 11.6 ± 1.8% vs. 6.3 ± 0.7%, *P *= .013) and ROS level (Figure 3H,I; 1.4 ± 0.1‐fold, *P *= .03). These data indicate that Ara‐C treatment impaired both the cellular status and functions of BM EPCs. Although HSPG2 treatment led to no obvious differences in the quality or functions of BM EPCs in the CTL group (Figure 3A–I), we found that the Ara‐C‐impaired BM EPCs were repaired by the HSPG2 treatment, as demonstrated by the increased number of double‐positive‐stained cells (Figure 3A,B; 30.2 ± 3.4 vs. 20.0 ± 2.5, *P *= .001), restored tube formation ability (Figure 3C,D; 5621.0 ± 188.9 vs. 3298.0 ± 221.8, *P *= .0002), migration ability (Figure 3E,F; 161.8 ± 17.0 vs. 100.2 ± 10.4, *P *= .0007), decreased level of cell apoptosis (Figure 3G; 7.7 ± 1.3% vs. 11.6 ± 1.8%, *P *= .003) and decreased ROS level (Figure 3H,I; 1.2 ± 0.1‐fold vs. 1.4 ± 0.1‐fold, *P *= .006). These results uncovered that the HSPG2 treatment can reverse the chemotherapy‐induced BM EPC damage in vitro.

### 3.4 HSPG2 recovered the impaired haematopoiesis‐supporting ability in BM EPCs from HCs caused by Ara‐C

**FIGURE 4:**
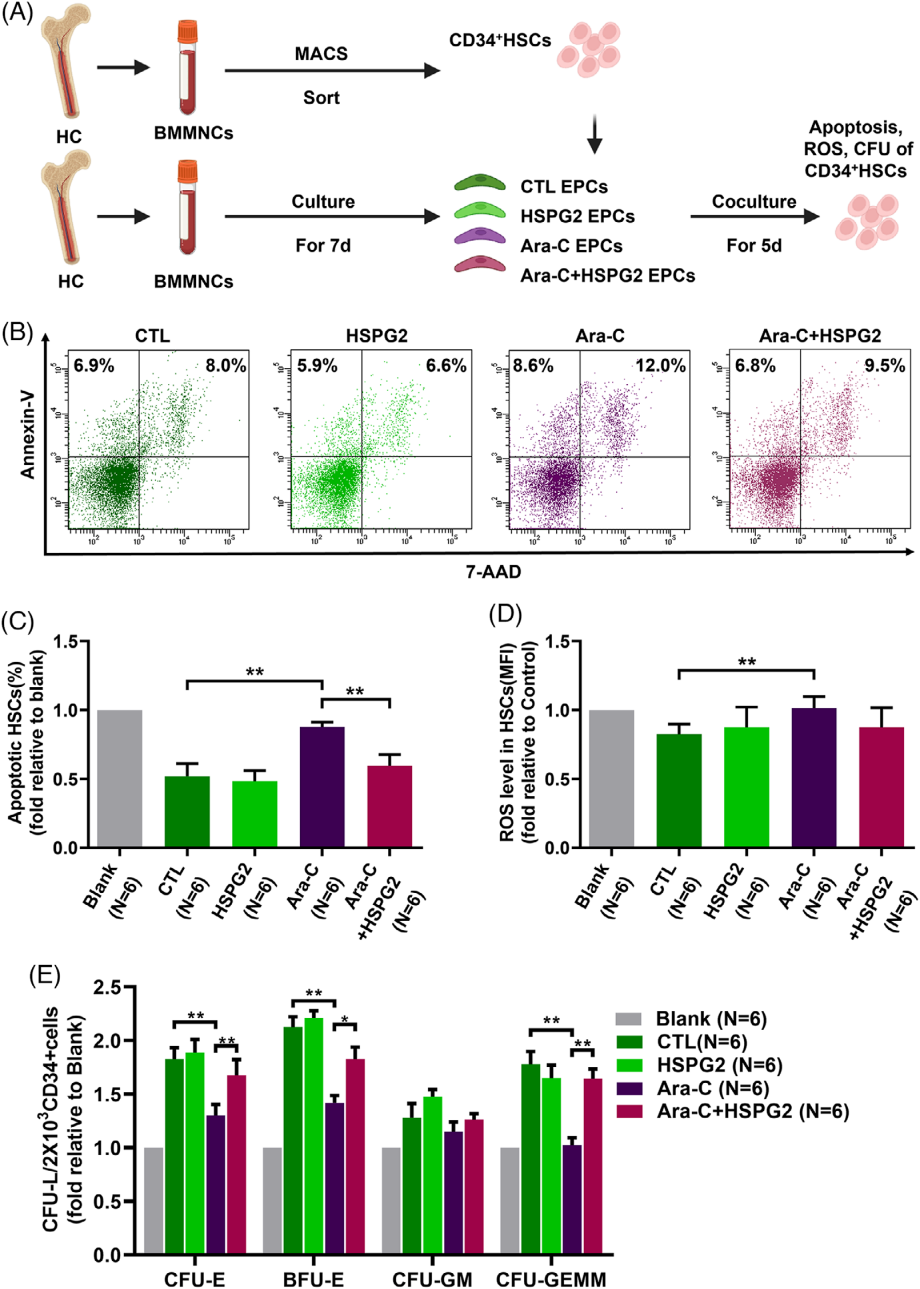
HSPG2 recovered the impaired haematopoiesis‐supporting ability in BM EPCs from HCs caused by Ara‐C. To investigate the effects of chemotherapy and HSPG2 on the haematopoiesis‐support ability of BM EPCs, we divided 7‐day cultivated BM EPCs from HCs into the following groups for Ara‐C and HSPG2 intervention in vitro: the control (CTL) group, the HSPG2 intervention (HSPG2) group, the Ara‐C intervention (Ara‐C) group, and the HSPG2+Ara‐C intervention (Ara‐C+HSPG2) group. (A) Schematic diagram showing the study design. Representative flow cytometry images (B) and quantification (C) of the ratio of apoptotic HSCs after 5 days of coculture with BM EPCs in each group are shown. (D) Quantification of the ROS levels (MFIs) of HSCs after coculture. (E) CFU formation efficacies of HSCs, including CFU‐E, BFU‐E, burst forming unit‐erythroid; CFU‐GM and CFU‐GEMM, after coculture with BM EPCs from HCs in each group. The data represent the mean ± SEM. *P ≤ .05, **P ≤ .01. BM EPC, Bone marrow endothelial progenitor cell; HC, healthy control; HSC, haematopoietic stem cell; CFU, colony‐forming unit.

To investigate the effects of chemotherapy on the haematopoiesis‐supporting ability of BM EPCs, we performed the BM EPC‐CD34^+^ cell coculture assays. After coculture for 5 days, the percentage of apoptotic cells, the level of ROS and the CFU efficiencies of CD34^+^ cells were determined. Consistent with the results of the functional assays, the Ara‐C treatment severely damaged the haematopoiesis‐supporting ability of BM EPCs, as measured by the significantly increased level of apoptosis rate (Figure 4B,C; 0.9% ± 0.04%‐fold vs. 0.5% ± 0.09%‐fold, *P *= .005) and ROS level (Figure 4D; 1.0 ± 0.08‐fold vs. 0.8 ± 0.07‐fold, *P *= .001), and decreased CFU efficacy of CD34^+^ cells (Figure 4E): colony forming unit‐erythroid (CFU‐E) (1.3 ± 0.1‐fold vs. 1.8 ± 0.1‐fold, *P *= .001), burst forming unit‐erythroid (BFU‐E) (1.4 ± 0.07‐fold vs. 2.1 ± 0.09‐fold, *P *= .004), and colony forming unit‐erythroid, granulocyte‐macrophage and megakaryocyte (CFU‐GEMM) (1.0 ± 0.07‐fold vs. 1.8 ± 0.11‐fold, *P *= .003).

The HSPG2 treatment reversed the impaired haematopoiesis‐supporting ability caused by Ara‐C, with a notably decreased level of the apoptosis rate (Figure 4B,C; 0.6% ± 0.08%‐fold vs. 0.9% ± 0.04%‐fold, *P *= .004), the decreased ROS level and significantly increased CFU efficacy of CD34^+^ cells (Figure 4E): CFU‐E (1.7 ± 0.1‐fold vs. 1.3 ± 0.1‐fold, *P *= .01), BFU‐E (1.8 ± 0.11‐fold vs. 1.4 ± 0.07‐fold, *P *= .02), CFU‐GEMM (1.6 ± 0.09‐fold vs. 1.0 ± 0.07‐fold, *P *= .007). These results indicate that the HSPG2 treatment can restore the haematopoiesis‐supporting ability of BM EPCs damaged by Ara‐C in vitro.

### 3.5 HSPG2 repaired BM EPC function and improved the haematopoiesis‐supporting ability of BM EPCs from AML‐CR patients

**FIGURE 5:**
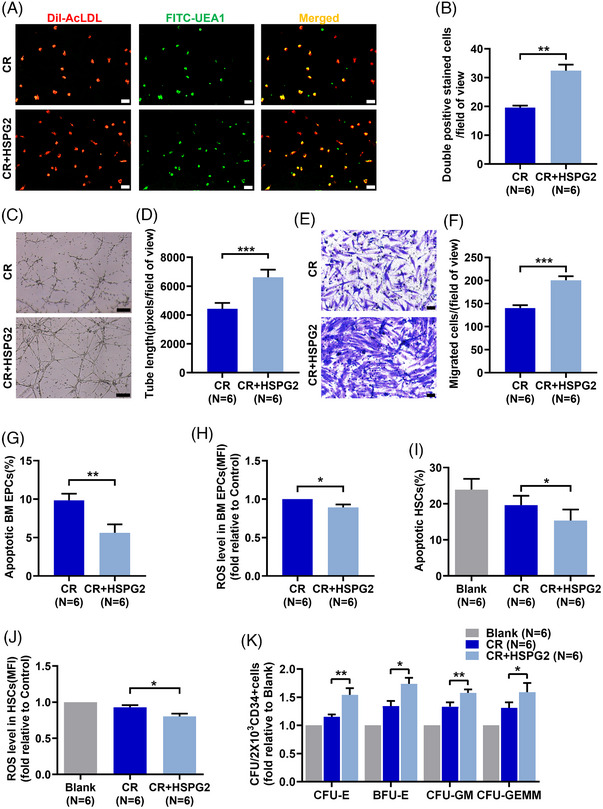
HSPG2 repaired BM EPC function and improved their haematopoiesis‐supporting ability in AML‐CR patients. To investigate the effects of HSPG2 on the functions and haematopoiesis‐supporting ability of CR EPCs, we divided 7‐day cultivated BM EPCs into the following groups for HSPG2 intervention in vitro: the CR EPCs without HSPG2 intervention (CR) group and the CR EPCs with HSPG2 intervention (CR+HSPG2) group. Representative images (scale bars represent 50 µm) (A) and quantification (B) of double‐positive BM EPCs stained with DiI‐AcLDL (red) and FITC‐UEA I (green) (merged in yellow)/field of view (original magnification, 10×). Representative images (scale bars represent 200 µm) (C) and quantification (D) of tube formation (pixels of tubes per field of view) by BM EPCs (original magnification, 4×). Representative images (scale bars represent 50 µm) (E) and quantification (F) of migrated cells (original magnification, 10×). (G) Quantification of the percentage of apoptotic BM EPCs. (H) Quantification of the level of intracellular ROS in BM EPCs. (I) Quantification of the percentage of apoptotic HSCs after coculture with BM EPCs. (J) Quantification of the level of intracellular ROS in HSCs. (K) CFU formation efficacies of HSCs, including CFU‐E, BFU‐E, burst forming unit‐erythroid; CFU‐GM and CFU‐GEMM, after coculture with BM EPCs. The data represent the mean ± SEM. *P ≤ .05, **P ≤ .01, ***P ≤ .001. AML, acute myeloid leukaemia; BM EPC, Bone marrow endothelial progenitor cell; CFU, colony‐forming unit; CR, complete remission; Dil‐Ac‐LDL, Dil‐acetylated low‐density lipoprotein; FITC‐UEA1, FITC‐labelled ulex europaeus agglutinin I; HSC, haematopoietic stem cell; ROS, reactive oxygen species.

In vitro treatment with HSPG2 improved the number of double‐positive stained cells (Figure 5A,B; 32.4 ± 2.1 vs. 19.6 ± 0.7, *P *= .001), the tube formation ability (Figure 5C,D; 6615.0 ± 529.1 vs. 4426.0 ± 413.5, *P *= .0008) and the migration capacity (Figure 5E,F; 200.3 ± 8.7 vs. 140.2 ± 6.2, *P *= .0003) of CR EPCs. Moreover, the HSPG2 treatment decreased the cell apoptosis rate (Figure 5G; 5.6% ± 1.1% vs. 9.9% ± 0.9%, *P *= .002) and the ROS level (Figure 5H; 0.9 ± 0.04‐fold, *P *= .037) of CR EPCs. The HSPG2 treatment promoted the haematopoiesis‐supporting ability of CR EPCs, as measured by notably decreased level of the apoptosis rate (Figure 5I; 15.4% ± 3.03% vs. 19.6% ± 2.57%, *P *= .035), decreased ROS level (Figure 5J; 0.8 ± 0.04‐fold vs. 0.9 ± 0.03‐fold, *P *= .040) of CD34^+^cells enhanced CFU efficacy of CD34^+^cells(Figure 5K), including CFU‐E (1.5 ± 0.12‐fold vs. 1.2 ± 0.04‐fold, *P *= .009), BFU‐E (1.7 ± 0.11‐fold vs. 1.3 ± 0.09‐fold, *P *= .036), colony forming unit‐granulocyte and macrophage (CFU‐GM) (1.6 ± 0.06‐fold vs. 1.3 ± 0.07‐fold, *P *= .007) and CFU‐GEMM (1.6 ± 0.16‐fold vs. 1.3 ± 0.10‐fold, *P *= .018). The results above proved that the reduced haematopoiesis‐supporting ability of CR EPCs could be restored by the HSPG2 treatment in vitro.

### 3.6 HSPG2 did not increase the leukaemia cell‐supporting ability of BM EPCs from AML‐CR patients

**FIGURE 6:**
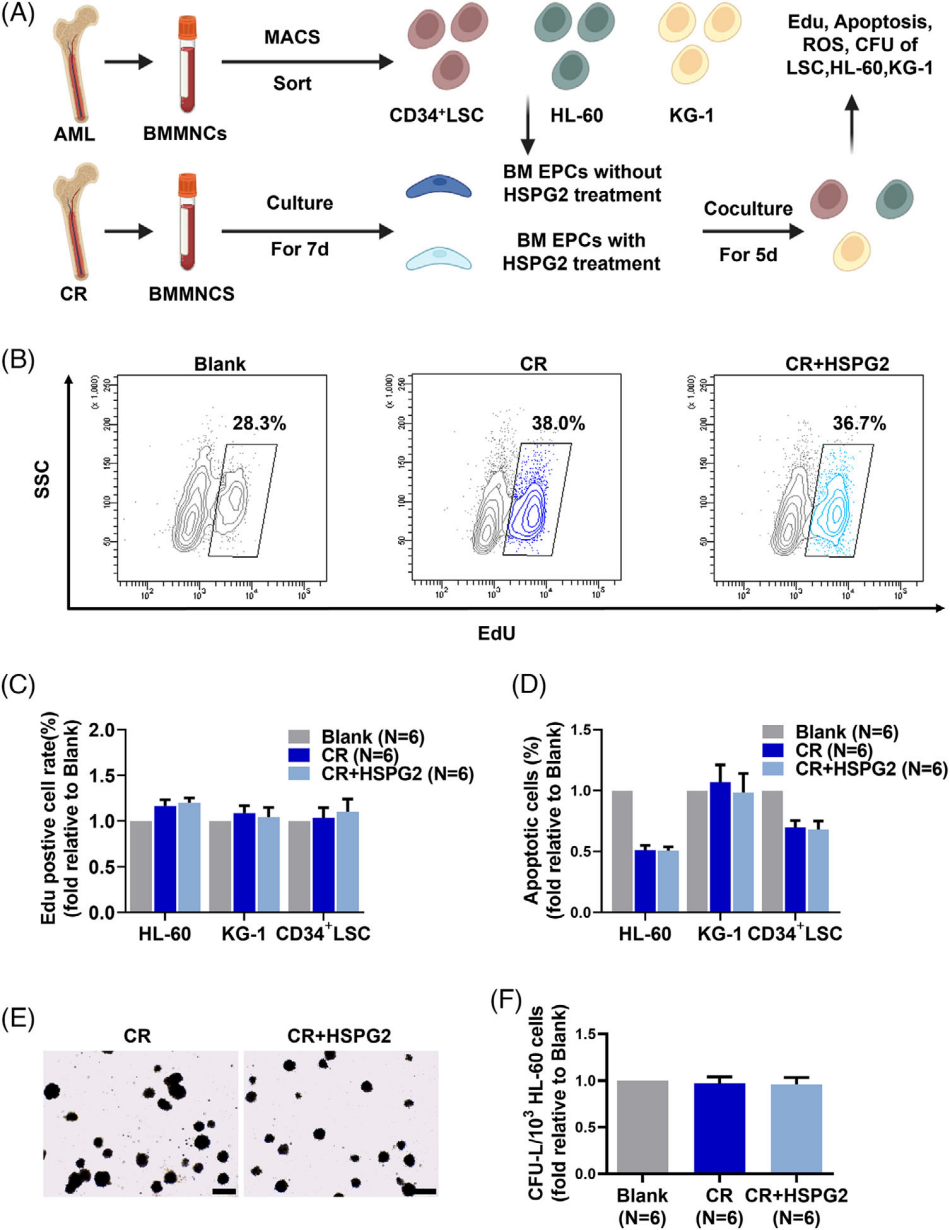
HSPG2 did not increase the leukaemia cell‐supporting ability of BM EPCs from AML‐CR patients. To investigate the effects of HSPG2 on the ability of CR EPCs to support leukaemia cells, we divided CR EPCs into the following groups and then cocultured them with HL‐60 cells, KG‐1 cells, and CD34^+^ cells from AML patients: the CR EPCs without HSPG2 intervention (CR) group and the CR EPCs with HSPG2 intervention (CR+HSPG2) group. (A) Schematic diagram of the study design. Representative images (B) and quantification of the EdU‐positive rates (C) of HL‐60 cells, KG‐1 cells, and CD34^+^ cells from AML patients. (D) Quantification of the apoptosis ratios of HL‐60 cells, KG‐1 cells, and CD34^+^ cells from AML patients. Representative images (scale bars represent 200 µm) (E) and quantification (F) of the CFU‐L plating efficacies of HL‐60 cells after coculture with BM EPCs (original magnification, 4×). Three power fields were randomly selected and used for counting, and the results were averaged for each sample. The data represent the mean ± SEM. BM EPC, Bone marrow endothelial progenitor cell; CFU, colony‐forming unit; CR, complete remission; AML, acute myeloid leukaemia; EdU, ethynyl‐deoxyuridine.

To explore the role of HSPG2 on malignant haematopoiesis, we used a series of in vitro coculture assays, and a schematic diagram of the experiment plan is provided (Figure 6A). Importantly, the HSPG2 treatment did not increase the leukaemia cell‐supporting ability of CR EPCs, which specifically manifested as no significant increase in cell proliferation ability (Figure 6B,C), no significant decrease in cell apoptosis (Figure 6D) and no significant changes in the CFU‐L formation ability (Figure 6E,F). These findings indicate that HSPG2 promotes the normal haematopoiesis‐supporting ability of CR EPCs (Figure 5K), without affecting their leukaemia cell‐supporting ability (Figure 6F).

### 3.7 RNA‐seq analysis revealed the internal changes in HSPG2 knockdown BM EPCs from HCs

**FIGURE 7:**
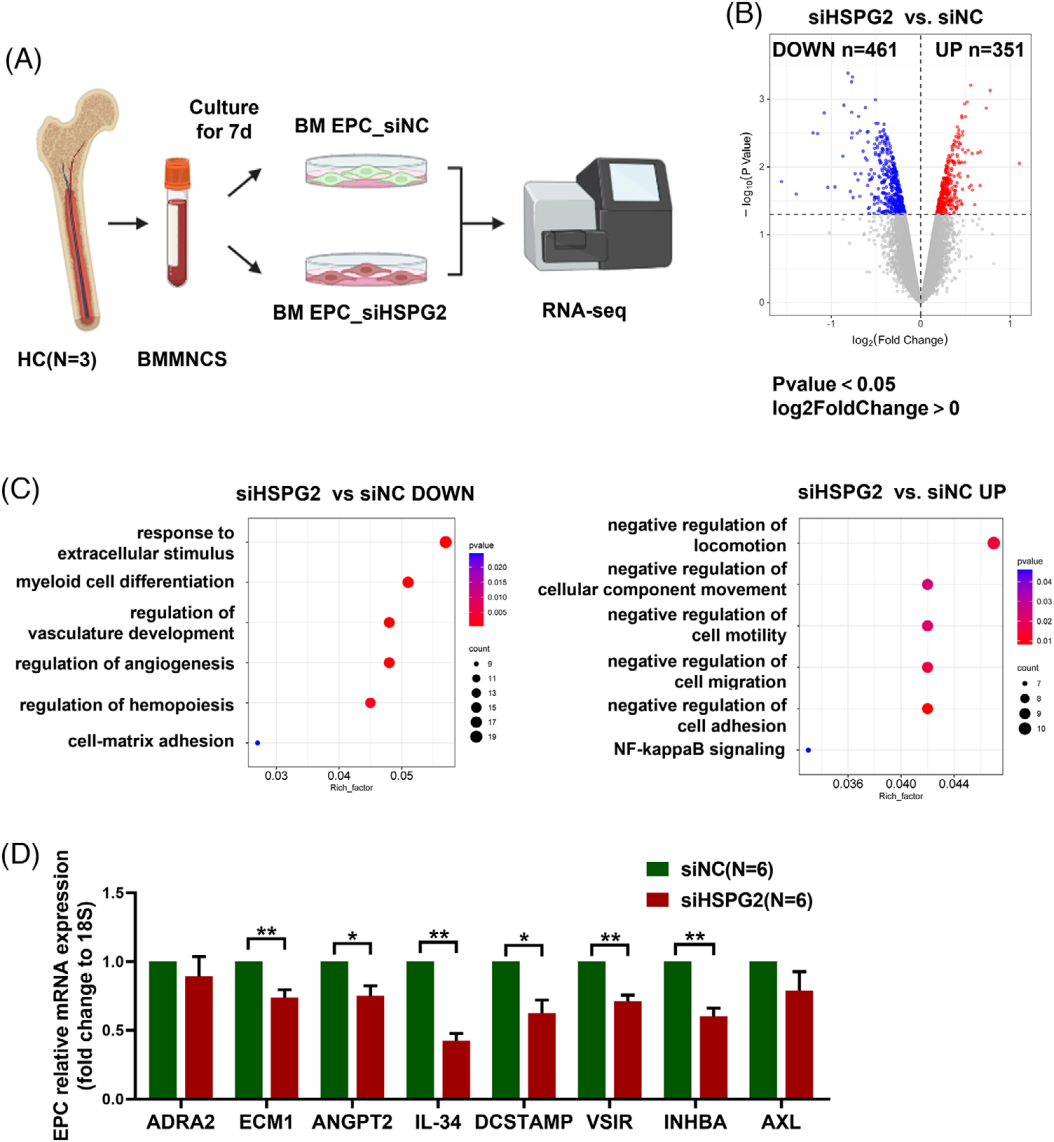
RNA‐seq analysis revealed the internal changes in HSPG2 knockdown BM EPCs from HCs. (A) Schematic diagram of the RNA‐seq analysis. (B) Volcano plot showing the genes whose expression was upregulated (red) or downregulated (blue) in BM EPCs in the siHSPG2 group. *P*‐value < .05 and |Log2(FoldChange)| > 0. (C) Bubble diagram showing decreased (left) and increased (right) GOBP terms for differentially expressed genes. (D) The relative mRNA levels of the *ADRA2*, *ECM1*, *ANGPT2*, *IL‐34*, *DCSTAMP*, *VSIR*, *INHBA* and *AXL* genes in HC‐EPCs before and after *HSPG2* knockdown were determined via qRT‐PCR. The relative mRNA analyses were performed via the Wilcoxon matched‐pairs signed rank test. The data represent the mean ± SEM. *P ≤ .05, **P ≤ .01. BM EPC, Bone marrow endothelial progenitor cell; HC, healthy control; GOBP, Gene Ontology biological process.

To explore the possible mechanism, RNA‐seq was conducted and a schematic diagram of our experimental plan is provided (Figure 7A). Amongst the 17761 genes, 351 were upregulated whilst 461 were downregulated in the siHSPG2 group (Figure 7B). Moreover, DGEs were enriched in GOBP terms, as shown in the bubble diagram: myeloid cell differentiation, regulation of angiogenesis and regulation of haemopoiesis were decreased, whereas negative regulation of cell migration, locomotion and adhesion were increased in the BM EPCs of the siHSPG2 group (Figure 7C).

To further verify, we validated the downregulated genes associated with these biological behaviours through qPCR. The mRNA expression levels of endothelial cell functional genes, including *ADRA2*, *ECM1* and haematopoietic regulation related genes, including *ANGPT2*, *IL‐34*, *DCSTAMP*, *VSIR*, *INHBA* and *AXL*, were decreased in the EPCs of the siHSPG2 group (Figure 7D), which are in accordance with our in vitro experiments. The above results confirmed that HSPG2 is a key molecule in the regulation of EPC functions.

## 4 DISCUSSION

**FIGURE 8:**
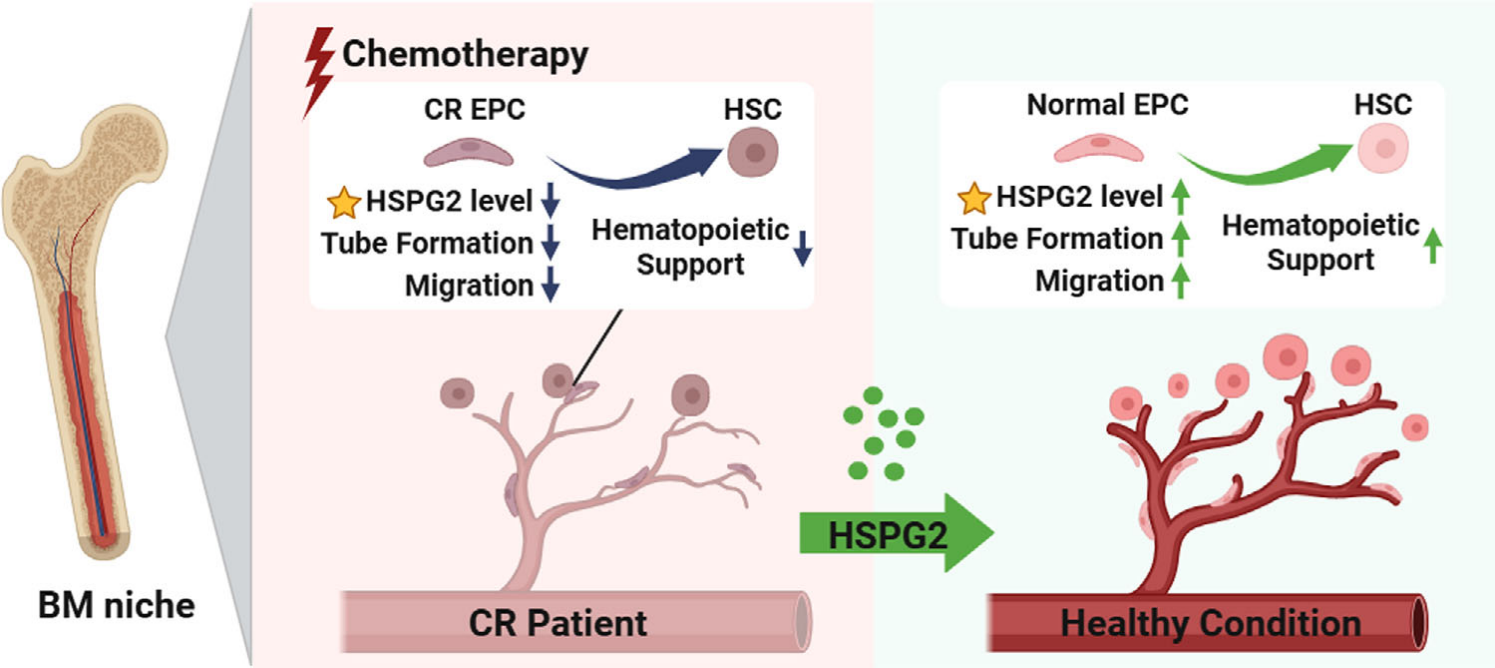
The HSPG2 level in the BM EPCs of CR patients was decreased, which was related to the BM EPC dysfunction. HSPG2 treatment restored the BM EPC function of CR patients without affecting their leukaemia cell‐supporting ability. BM EPC, Bone marrow endothelial progenitor cell; CR, complete remission.

This study demonstrated the important effect of HSPG2 in promoting the BM EPC function of AML‐CR patients after chemotherapy. According to *HSPG2* knockdown assays, we found that the impaired BM EPC functions of AML‐CR patients were associated with their decreased HSPG2 levels. Moreover, the positive effects of HSPG2 on BM EPCs were confirmed by a series of in vitro studies. Mechanistically, RNA‐seq combined with qPCR validation revealed that the BM EPC function and haematopoietic regulation‐related genes were significantly decreased after knockdown of the *HSPG2* gene. Our findings may suggest a potential treatment strategy for AML‐CR patients after chemotherapy (Figure 8).

Haematopoietic dysfunction is a serious side effect of chemotherapy, patients suffer from a series of complications, such as anaemia, haemorrhage and even fatal infections.^35^, ^36^ Well‐functioning BM EPCs, which are critical BM microenvironment components involved in haematopoietic regulation, participate in the development, homeostasis, self‐renewal and differentiation of HSCs.^37^ Hooper et al. reported that inhibition of VEGFR2 signalling in wild‐type mice severely impaired EC reconstruction and prevented haematopoietic reconstitution.^38^ Our previous studies have revealed that impaired BM EPCs participate in the occurrence of haematopoietic dysfunction related diseases, whereas repairing dysfunctional BM EPCs could promote HSC functions and thus alleviate the diseases.^14^, ^16^, ^19^, ^23^, ^32^, ^33^ Therefore, it is important to identify the underlying mechanism and repair target for promoting BM EPC function in AML‐CR patients.

HSPG2, a large multidomain ECM proteoglycan, which is the key molecule in the ECM, plays an important role in cartilage formation, cell adhesion and tissue regeneration.^26^, ^39^, ^40^ HSPG2 can be secreted into the ECM by various cells including EPCs.^41^ In turn, HSPG2 in the ECM regulates cell proliferation and differentiation by binding to cell membrane surface proteins.^27^, ^28^ Ishijima et al. reported that HSPG2 promoted vascular invasion of EPCs in HSPG2^−^/^−^ mice by upregulating the VEGF receptor pathway.^30^ For haematopoiesis, Grigorian et al. reported that in *Drosophila*, a mutation in *HSPG2* leads to impaired haematopoietic cell maturation.^31^ However, the regulatory role of HSPG2 on the functions of human BM EPCs is unidentified.

Here, the effect of HSPG2, especially in BM EPCs, was investigated via a *HSPG2* knockdown assay and in vitro experiments. We found for the first time that the functions, especially the haematopoiesis‐support ability, of BM EPCs are related to their HSPG2 level. Ara‐C intervention impaired both the HSPG2 level and BM EPC's functions, whilst the HSPG2 treatment alleviated this damage in vitro. Importantly, we found that HSPG2 did not increase the leukaemia cells‐supporting ability of CR EPC.

RNA‐seq revealed that the regulation of angiogenesis and haemopoiesis was decreased, whereas the negative regulation of cell migration was increased in the BM EPCs of the siHSPG2 group. Further, qPCR results revealed that genes related to haematopoiesis and EPC function, such as *extracellular matrix protein 1* (*ECM1*), *angiopoietin 2* (*ANGPT2*), *Interleukin 34* (*IL‐34*), *dendritic cell‐specific transmembrane protein* (*DCSTAMP*), *v‐set immunoregulatory receptor* (*VSIR*), *inhibin subunit beta A*(*INHBA*) were significantly downregulated in BM EPCs from the siHSPG2 group.^42^, ^43^, ^44^, ^45^, ^46^ RNA‐seq results are aligned with experiments in vitro, suggesting that HSPG2 is a key molecule in regulating BM EPC function and their haematopoietic‐supporting capacity.

Nevertheless, there is still controversy regarding the relationship between HSPG2 and the prognosis of cancer patients. Grindel et al. reported that the high‐expression level of HSPG2 may be an indicator of invasion and distant metastasis in prostate cancer.^47^ Controversially, Zhang et al. found that HSPG2 mutation is predictive of a better immune checkpoint inhibitors response and promotes the survival outcome of melanoma and lung cancer.^48^ In haematological malignancy, Zhou et al. reported that HSPG2 overexpression in BMMNCs was related to poor prognosis in de novo AML patients.^49^ Different from the role of HSPG2 in AML cells,^49^ this study focused on the HSPG2 level on BM EPCs in AML‐CR patients after chemotherapy, we found that HSPG2 could promote the normal haematopoiesis‐supporting ability of BM EPCs without affecting their leukaemia cell‐supporting ability via a series of in vitro coculture assays. We are aware, however, although in vitro findings are compelling, further evidence based on animal models may provide stronger evidence of HSPG2's efficacy in restoring normal haematopoiesis in AML after chemotherapy in the future. Moreover, long‐term effects of HSPG2 on both normal haematopoiesis and leukaemia recurrence risk need to be further elucidated by long‐term monitoring in AML patients after chemotherapy.

## 5 CONCLUSION

In summary, this work demonstrated that HSPG2 can repair the BM EPC function in AML‐CR patients after chemotherapy without altering their leukaemia cell‐supporting ability. Our data may offer a future therapeutic approach not only for AML patients but also for patients with other cancers after chemotherapy.

## AUTHOR CONTRIBUTIONS

**Yuan Kong, Xiao‐Jun Huang**: Writing—review & editing & revision, supervision, project administration, funding acquisition, conceptualization. **Chen‐Yuan Li**: Collection and assembly of data, data analysis and manuscript writing & revision. **Zhen‐Kun Wang, Tong Xing**: Resources, methodology. **Meng‐Zhu Shen**, **Xin‐Yan Zhang**, **Dan‐Dan Chen**, **Yu Wang**, **Hao Jiang**, **Qian Jiang**: Resources.

## CONFLICT OF INTEREST STATEMENT

The authors declare no conflicts of interest.

## DATA AVAILABLITY STATEMENT

The data that support the findings of this study are available upon reasonable request from the corresponding author.

## ETHICS STATEMENT AND CONSENT TO PARTICIPATE

The study was conducted in accordance with the Declaration of Helsinki, and was approved by the Ethics Committee of Peking University People's Hospital. Informed consent was obtained from all subjects involved in the study.

## Supporting information



Supporting information
