# Supplementary material for: HSPG2 could promote normal haematopoiesis in acute myeloid leukaemia patients after complete remission by repairing bone marrow endothelial progenitor cells
Source: Clin Transl Med. 2025 Feb 6;15(2):e70220. doi: 10.1002/ctm2.70220 (PMC11802241; doi:10.1002/ctm2.70220)
Supplement: Supplementary file 1 — Supporting information [file CTM2-15-e70220-s001.docx]

**Supplementary Material**

**Headline: HSPG2 Could Promote Normal Hematopoiesis in Acute Myeloid Leukemia Patients after Complete Remission by Repairing Bone Marrow Endothelial Progenitor Cells**

**Authors:** Chen-Yuan Li^1^, Zhen-Kun Wang^1^, Tong Xing^1,2^, Meng-Zhu Shen^1^, Xin-Yan Zhang^1^, Dan-Dan Chen^1^, Yu Wang^1^, Hao Jiang^1^, Qian Jiang^1^, Xiao-Jun Huang^1,2^*, Yuan Kong^1^*

*Yuan Kong and Xiao-Jun Huang are co-corresponding authors.

**Correspondence to:**Yuan Kong**:** [successky@163.](mailto:successky@163.)com

Xiao-Jun Huang**:** huangxiaojun@bjmu.edu.cn

**SUPPLEMENTAL TABLES**

**Supplemental Table 1. Characteristics of CR patients and HC**

| **Characteristics** | **HC (N=20)** | **CR-IA (N=20)** | **CR-HAA (N=20)** | ***P-*value***** | ***P-*value****** |
| --- | --- | --- | --- | --- | --- |
| **Physical variables** |  |  |  |  |  |
| Age (years)^a^ | 51 (31−56) | 51 (27−60) | 46 (25−56) | 0.96 | 0.09 |
| Gender, female/male | 9/11 | 9/11 | 11/9 | 1 | 0.75 |
| **BM cell counts** |  |  |  |  |  |
| BM Blasts (%) ^a^ | 0 | 0.45 (0.11-3.0) | 0.81 (0.11-1.57) | <0.0001 | <0.0001 |
| **Blood cell counts** |  |  |  |  |  |
| WBC count (×10^9^/L) ^a^ | 7.54 (4.91−9.50) | 4.09 (2.3−6.89) | 4.55 (2.01−5.49) | <0.0001 | <0.0001 |
| ANC (×10^9^/L) ^a^ | 3.85 (2.64−5.90) | 2.14 (0.99−3.87) | 2.64 (0.60−3.40) | <0.0001 | <0.0001 |
| Hemoglobin (g/L) ^a^ | 152 (129−165) | 115 (89−155) | 112 (78−138) | <0.0001 | <0.0001 |
| Platelet (×10^9^/L) ^a^ | 228 (153−328) | 127(75−206) | 158 (81−226) | <0.0001 | <0.0001 |

**^a^** Data are reported as median (range).

* *P-*values present the comparison between the CR patients who received (IA) regimens and healthy control groups. The counts were compared using the Wilcoxon matched-pairs signed rank test. The criterion for statistical significance was P < 0.05.

** *P-*values present the comparison between CR who received homoharringtonine, aclarubicin, cytarabine (HAA) regimens as first induction chemotherapy patients and healthy control groups. The counts were compared using the Wilcoxon matched-pairs signed rank test. The criterion for statistical significance was P < 0.05.

**Abbreviations:** CR, complete remission; HC, healthy control; BM, bone marrow; WBC, white blood cell; ANC, absolute neutrophil count.

**Supplemental Table 2. Antibody information**

| Antibody name | | Provider | Catalog number |
| --- | --- | --- | --- |
| CD45-V500 | | Becton Dickinson | 560777 |
| CD34-FITC | | Biolegend | 343504 |
| CD309-PE | | Becton Dickinson | 560494 |
| HSPG2-AF647 | | Becton Dickinson | 565782 |
| CD34-PerCP | Biolegend | | 343522 |
| 7AAD | Becton Dickinson | | 559925 |
| Annexin V-APC | Biolegend | | 640920 |

**Supplemental Table 3. Primer sequence of genes**

| **Name** | **Forward primer** | **Reverse primer** |
| --- | --- | --- |
| *18S* | GTAACCCGTTGAACCCCATT | CCATCCAATCGGTAGTAGCG |
| *ADRA2* | TCGTCATCATCGCCGTGTTC | AAGCCTTGCCGAAGTACCAG |
| *ECM1* | AGCACCCCAATGAACAGAAGG | CTGCATTCCAGGACTCAGGTT |
| *ANGPT2* | CGAGTCATCGTATTCGAGCGG | CGAGTCATCGTATTCGAGCGG |
| *IL-34* | AAGGTGGAATCCGTGTTGTCC | AGCTTTGTTTACAGCAGGAGC |
| *DCSTAMP* | CGCTGCCTCCTGGATTATCAC | AAGCTCTTTGCCCTTAGGTTG |
| *VSIR* | ACGCCGTATTCCCTGTATGTC | TTGTAGAAGGTCACATCGTGC |
| *INHBA* | CCTCCCAAAGGATGTACCCAA | CTCTATCTCCACATACCCGTTCT |
| *AXL* | GTGGGCAACCCAGGGAATATC | GTACTGTCCCGTGTCGGAAAG |

**SUPPLEMENTAL FIGURES**

**Supplemental Figure1**

**
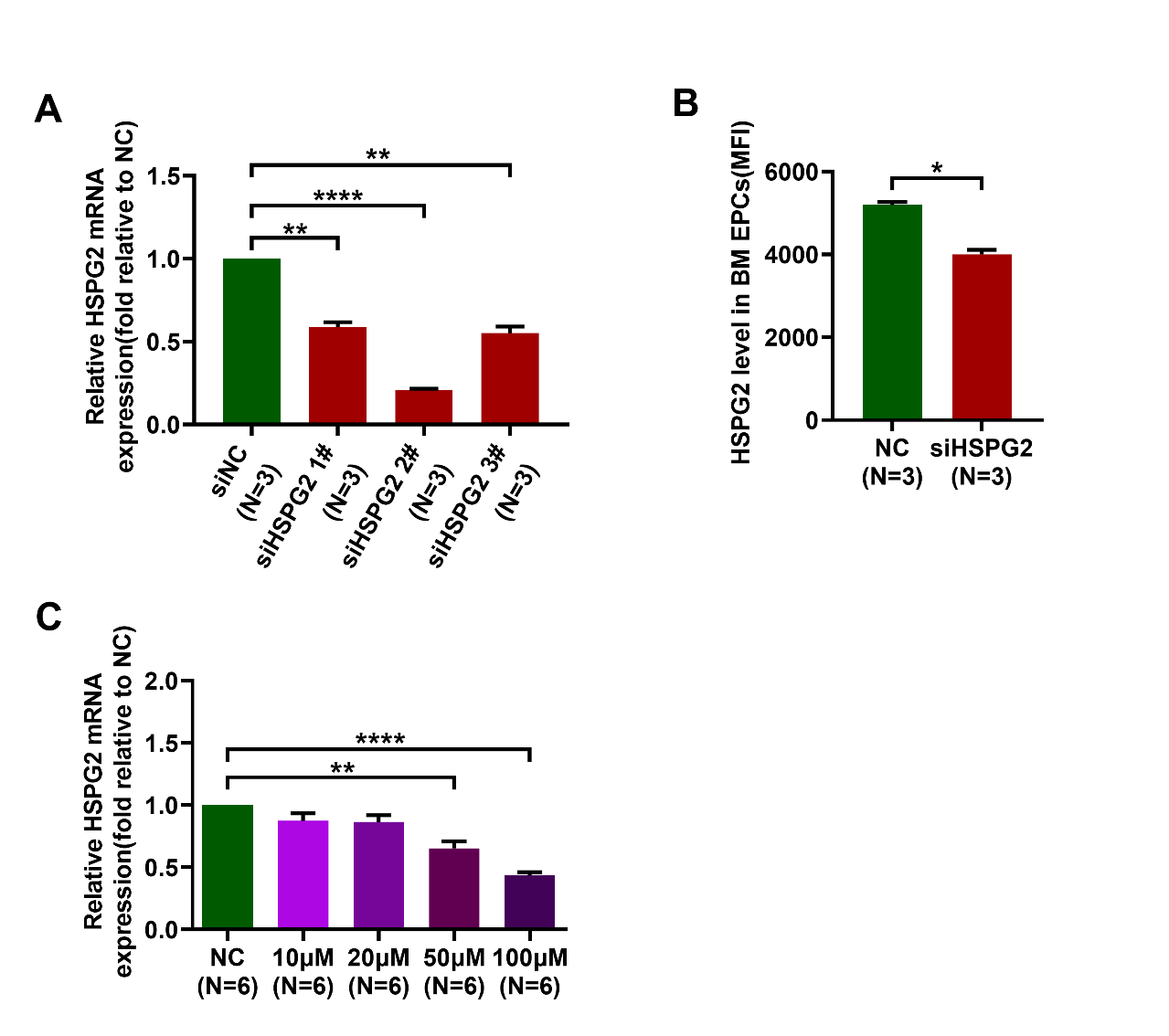
**

**Supplemental Figure1. The impact of transfection and chemotherapy on HSPG2 level. (A)** The relative HSPG2 mRNA expression levels of BM EPCs from each group of sequence. The knockdown efficiency of the 2^#^ sequence was the highest, so we used the 2^#^ sequence for subsequent experiments. **(B)** The HSPG2 expression levels of BM EPCs from negative control siRNA (NC) group and siHSPG2 siRNA (siHSPG2) group were analyzed by flow cytometry. **(C)** The relative HSPG2 mRNA expression levels of BM EPCs from each group of different concentrations of Ara-C.
